# Supplementary material for: Broadly neutralizing human monoclonal antibodies against BK polyomavirus genotypes
Source: J Gen Virol. 2025 Dec 22;106(12):002197. doi: 10.1099/jgv.0.002197 (PMC12721715; doi:10.1099/jgv.0.002197)
Supplement: Uncited Table S1. [file jgv-106-02197-s002.pdf]

Table S1: Summary of broadly neutralizing BKV clonotype mAbs

Clone	Heavy Chain (CDR3)		Heavy D Gene	Heavy J Gene	Clonotype	Fusion
	Length	Seq				
2B9	14	DPYNWNHGVYG MDV	IGHV3-23	IGHJ6	A	1
2F11*	10	HARSWNYVAY	IGHV5-51	IGHJ4	B	2
3D6*	10	HARSWNYVAY	IGHV5-51	IGHJ4	B	
4F1	10	HARNWNNVAY	IGHV5-51	IGHJ4	C	
2G2*	12	DRFLEWVEGFDP	IGHV3-23	IGHJ5	D	3
14F5*	12	DRFLEWVEGFDP	IGHV3-23	IGHJ5	D	
15C10*	12	DRFLEWVEGFDP	IGHV3-23	IGHJ5	D	
9G9	12	DRFLEWVEGFDS	IGHV3-23	IGHJ5	D	
1H10	11	ERRDGHKIFDC	IGHV3-11	IGHJ4	E	4
2F7*	11	ERRDGHKIFDC	IGHV3-11	IGHJ4	E	
4F7	11	ERRDGHKIFDC	IGHV3-11	IGHJ4	E	
2H1	11	ERRDGHKIFDC	IGHV3-11	IGHJ4	E	
4F9	11	ERRDGHKIFDW	IGHV3-11	IGHJ4	E	
5A8*	11	ERRDGHKIFDY	IGHV3-11	IGHJ4	E	
7A4*	11	ERRDGHKIFDY	IGHV3-11	IGHJ4	E	
8D10*	11	ERRDGHKIFDY	IGHV3-11	IGHJ4	E	
5H5	11	ERRDGHKIFDW	IGHV3-11	IGHJ4	E	
7C4	11	ERRDGHKIFDW	IGHV3-11	IGHJ4	E	
7H8	10	DSSSWFSLHY	IGHV3-23	IGHJ4	F	
8A11	11	ERRSGHKIFDC	IGHV3-11	IGHJ4	E	
8F4	11	ERRDGHKIFDW	IGHV3-11	IGHJ4	E	
8H6	11	ERRDGHKIFDC	IGHV3-11	IGHJ4	E	
9B4	11	ERREGHKIFDF	IGHV3-11	IGHJ4	F	

*Identical clones, respectively
